# Supplementary material for: Discovery of small molecule agonists of the Relaxin Family Peptide Receptor 2
Source: Commun Biol. 2022 Nov 4;5:1183. doi: 10.1038/s42003-022-04143-9 (PMC9636434; doi:10.1038/s42003-022-04143-9)
Supplement: Supplementary file 2 — Supplementary Information [file 42003_2022_4143_MOESM2_ESM.pdf]

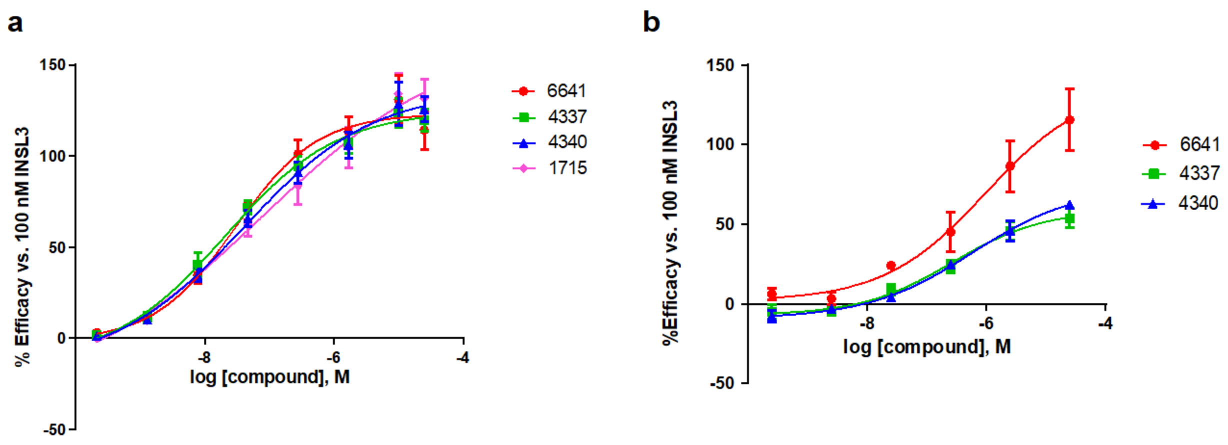

Supplementary Fig. 1: Compounds activate both human and mouse RXFP2 receptors. a Confirmatory CRE-Luciferase screening of RXFP2 agonists in stably transfected HEK-CRE-Luc-RXFP2 cells. **b** Activation of mouse RXFP2 receptor by the compounds in transiently transfected HEK293T-CRE-Luc cells. Compound response was normalized to 100 nM INSL3 as 100% efficacy and DMSO vehicle as 0% efficacy. Results are expressed as mean ± SEM of 3 independent experiments.

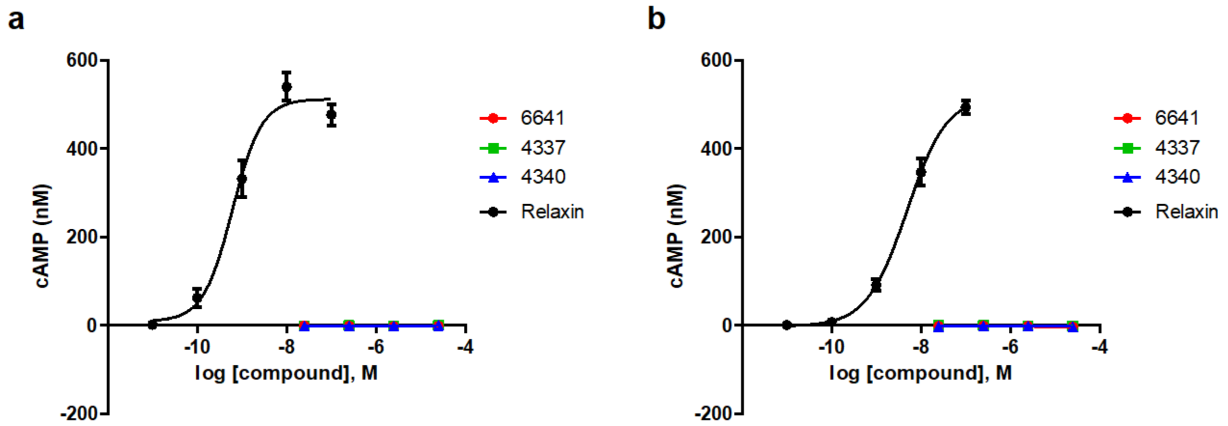

Supplementary Fig. 2 Compounds do not activate human or mouse RXFP1 receptors. HTRF cAMP counter-screen of RXFP2 agonists in HEK293T cells expressing **a** human RXFP1 and **b** mouse RXFP1. Relaxin 2 was used as positive control for RXFP1 receptor activation. Results are expressed as mean ± SEM of 3 independent experiments.

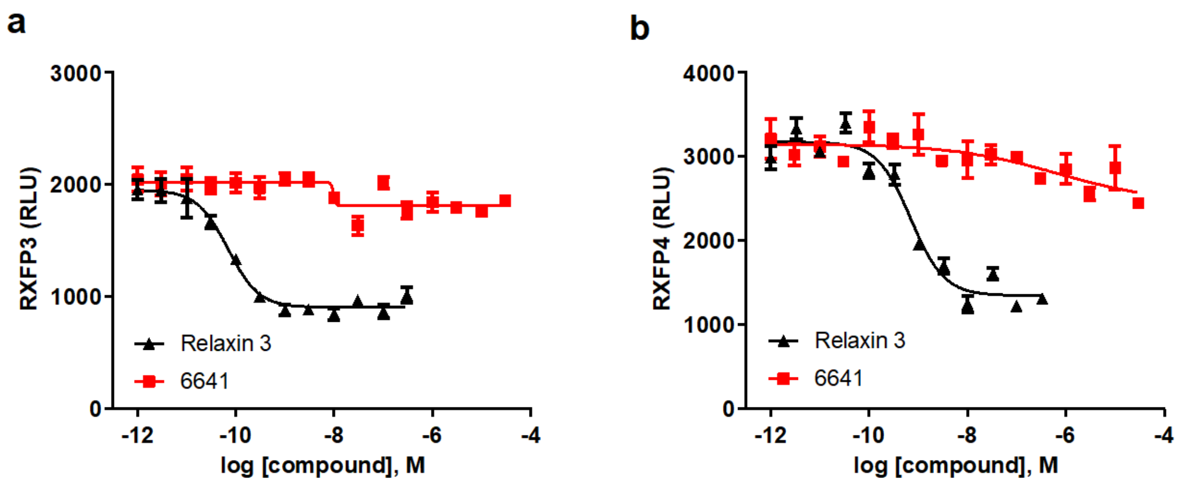

Supplementary Fig. 3: Compound 6641 does not activate human RXFP3 or RXFP4 receptors. cAMP production in HEK293T cells transiently transfected with human **a** RXFP3 and **b** RXFP4 receptors was induced by treatment with 0.1 μM of isoproterenol. cAMP inhibition was induced by Relaxin 3 but not by 6641. Results are reported as raw RLU values and represent the mean ± SEM of 4 technical replicates.

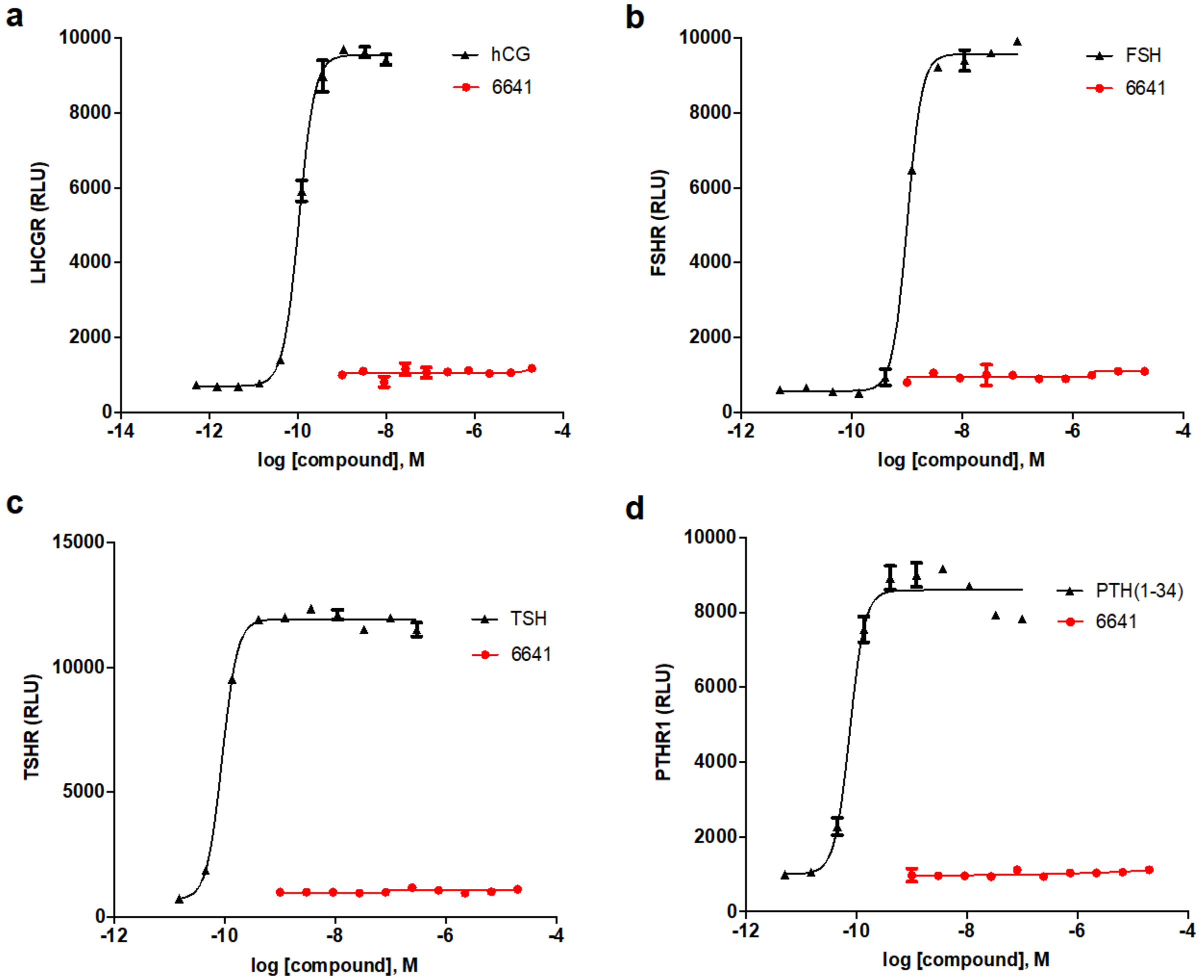

Supplementary Fig. 4: Compound 6641 does not activate human LHCGR, FSHR, TSHR, and PTHR1 receptors. cAMP activity was induced by corresponding recombinant hormones, whereas no changes in cAMP were detected when the cells were treated with 6641. Results are reported as raw RLU values and represent the mean ± SEM of 2 technical replicates.

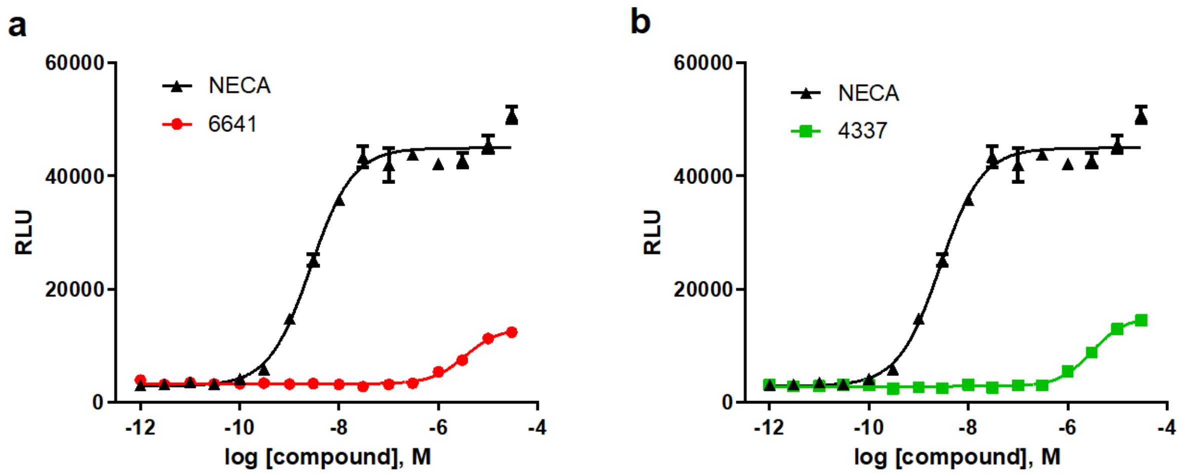

Supplementary Fig. 5: Compounds 6641 and 4337 induce β-arrestin binding to human ADORA1. β-arrestin translocation activity induced by **a** 6641 and **b** 4337 in ADORA1 transiently transfected HTLA cells. NECA was used as positive control for ADORA1 receptor activation. Results are reported as raw RLU values and represent the mean ± SEM of 4 technical replicates.

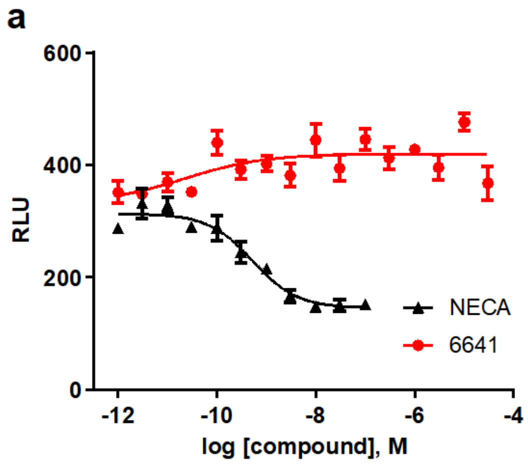

Supplementary Fig. 6: Compounds 6641 and 4337 do not inhibit cAMP through ADORA1 activation. cAMP in HEK293T cells transiently transfected with human ADORA1 was induced by treatment with 0.1 μM of isoproterenol. cAMP inhibition is shown by NECA but not by compounds **a** 6641 and **b** 4337. Results are reported as raw RLU values and represent the mean ± SEM of 4 technical replicates.

a

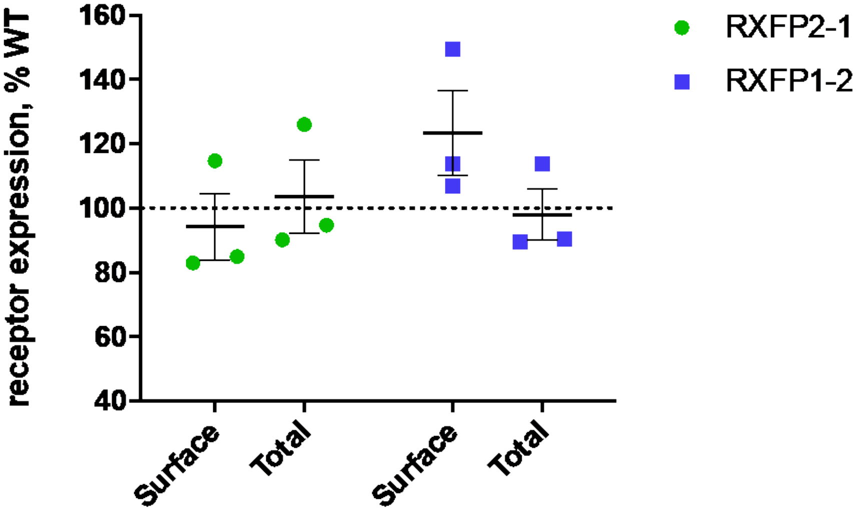

b

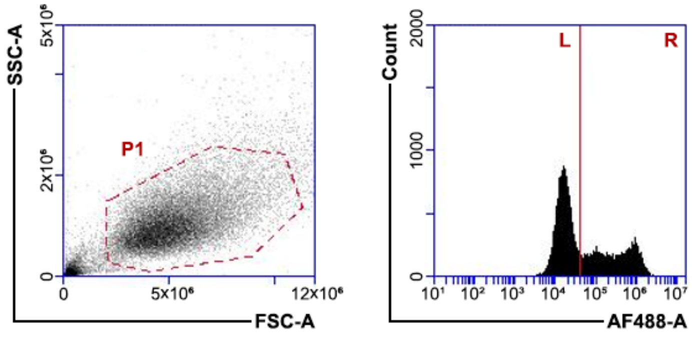

Supplementary Fig. 7: Chimeric and wild-type receptors are expressed at similar levels on the cell surface of transfected HEK293T cells. a Flow cytometry of FLAG-tagged receptors was analyzed for surface and total expression of the chimeric receptors and normalized to the respective WT receptor as 100% expression. Empty pcDNA3.1/Zeo(+) AmpR mammalian expression vector was used to establish background staining. Results are expressed as mean ± SEM of 3 independent experiments. **b** Representative example of gating strategy for transfected cells showing

the population analyzed (P1) and vertical gate delineating positive (R) and negative (L) cells. The vertical gate was set based on a mock-transfected control sample.

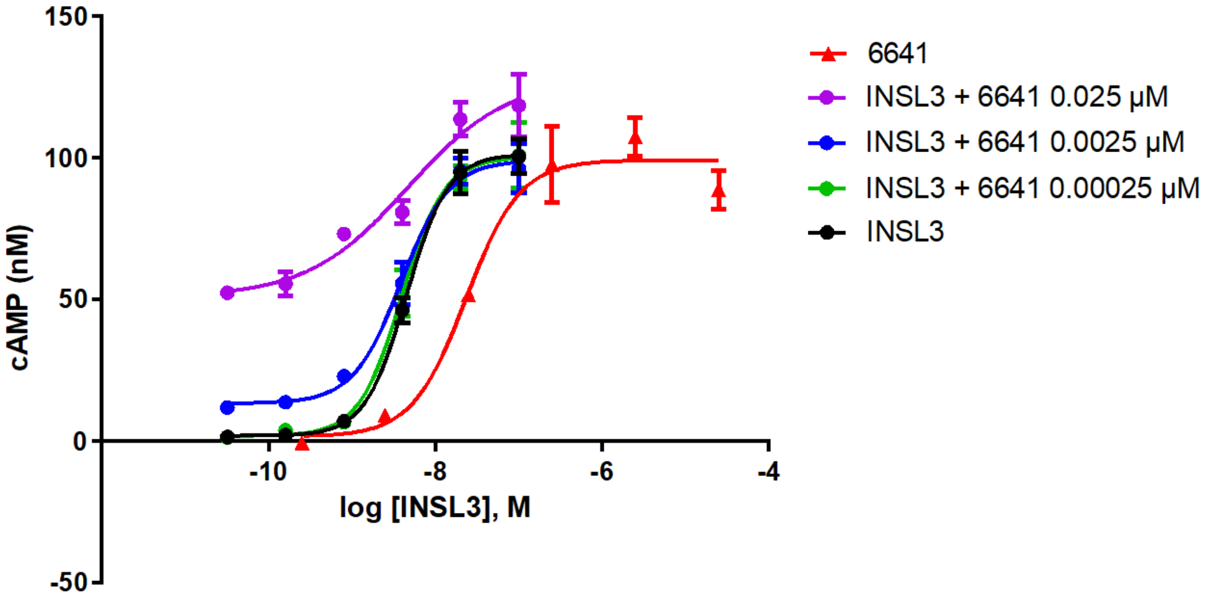

Supplementary Fig. 8: Compound 6641 is not an allosteric modulator of INSL3. cAMP response in HEK293T cells expressing human RXFP2 was measured by HTRF assay after treatment with a titration of INSL3 and suboptimal concentrations of 6641. Results are expressed as mean ± SEM of 3 independent experiments.

a

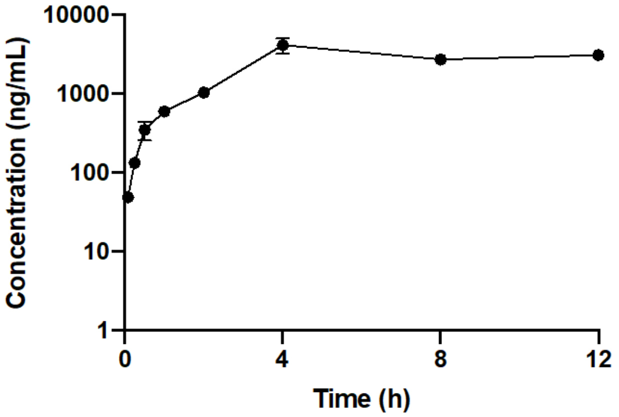

Route	Dosing Level (mg/kg)	Cl_obs (mL/min/kg)	$t_{1/2}$ (h)	t_{max} (h)	C_{max} (ng/mL)	AUC_{last} (h*ng/mL)	AUC_{Inf} (h*ng/mL)	AUC_%Extrap (%)	MRT (h)	AUC_{last}/D (h*mg/mL)	V_{ss}_obs (L/kg)	F (%)
IP	30	NA	19.2	4.00	4140	31791	117888	73.0	NA	1060	NA	NA

b

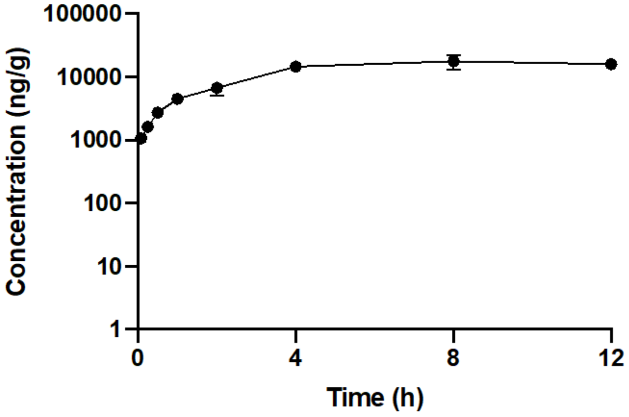

Route	Dosing Level (mg/kg)	t_{max} (h)	C_{max} (ng/g)	AUC_{last} (h*ng/g)	AUC_{Inf} (h*ng/g)	C_{max} Ratio (Liver/ Plasma)	AUC_{Last} Ratio (Liver/ Plasma)
IP	30	8.00	17773	161570	NA	4.29	5.08

c

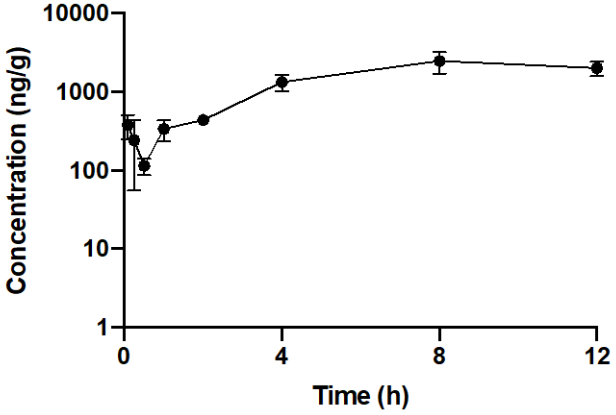

Route	Dosing Level (mg/kg)	t_{max} (h)	C_{max} (ng/g)	AUC_{last} (h*ng/g)	AUC_{Inf} (h*ng/g)	C_{max} Ratio (Bone/ Plasma)	AUC_{Last} Ratio (Bone/ Plasma)
IP	30	8.00	2456	18885	NA	0.593	0.594

Supplementary Fig. 9: Compound 6641 pharmacokinetic study after 30 mg/kg single dose IP administration. **a** Plasma, **b** liver and **c** bone profiles in female mice. The actual concentration (ng/g) is the detected value (ng/mL) multiplied by 4. Drug vehicle is 60% Phosal : 40% PEG300. 3 mice were used per time point. Results are expressed as the mean ± SEM.

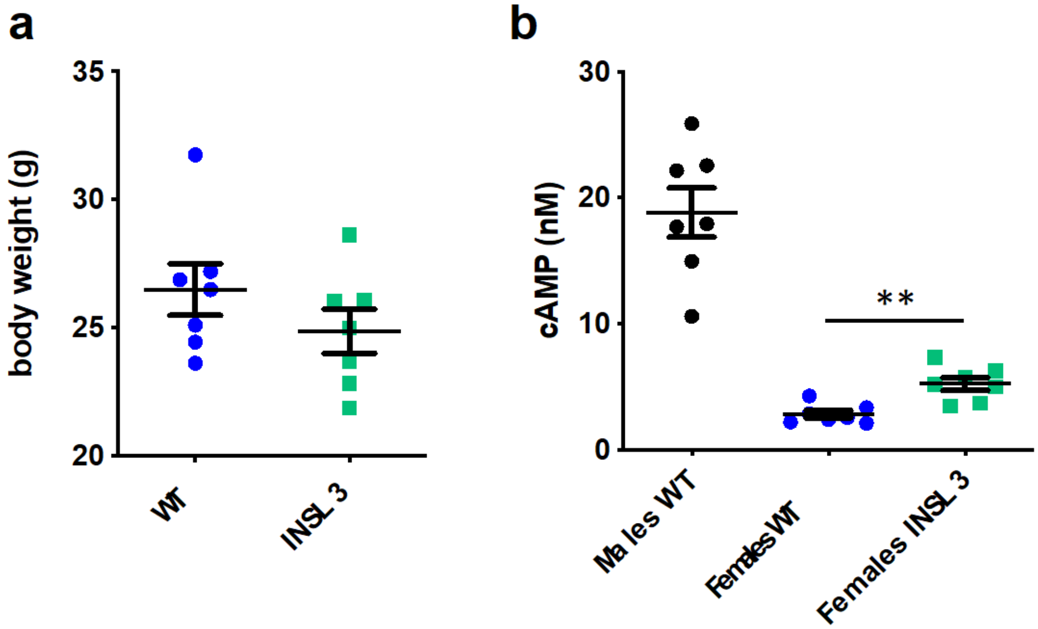

Supplementary Fig. 10 Validation of INSL3 transgenic mouse model. a *Insl3* overexpression does not affect body weight in female mice. **b** cAMP activity induced in HEK-RXFP2 cells by INSL3 present in serum from WT and INSL3 female mice. Serum from WT males was used as positive control. Results represent the mean ± SEM of 7 mice per group. **p<0.01 vs. WT females using Student's t-test.

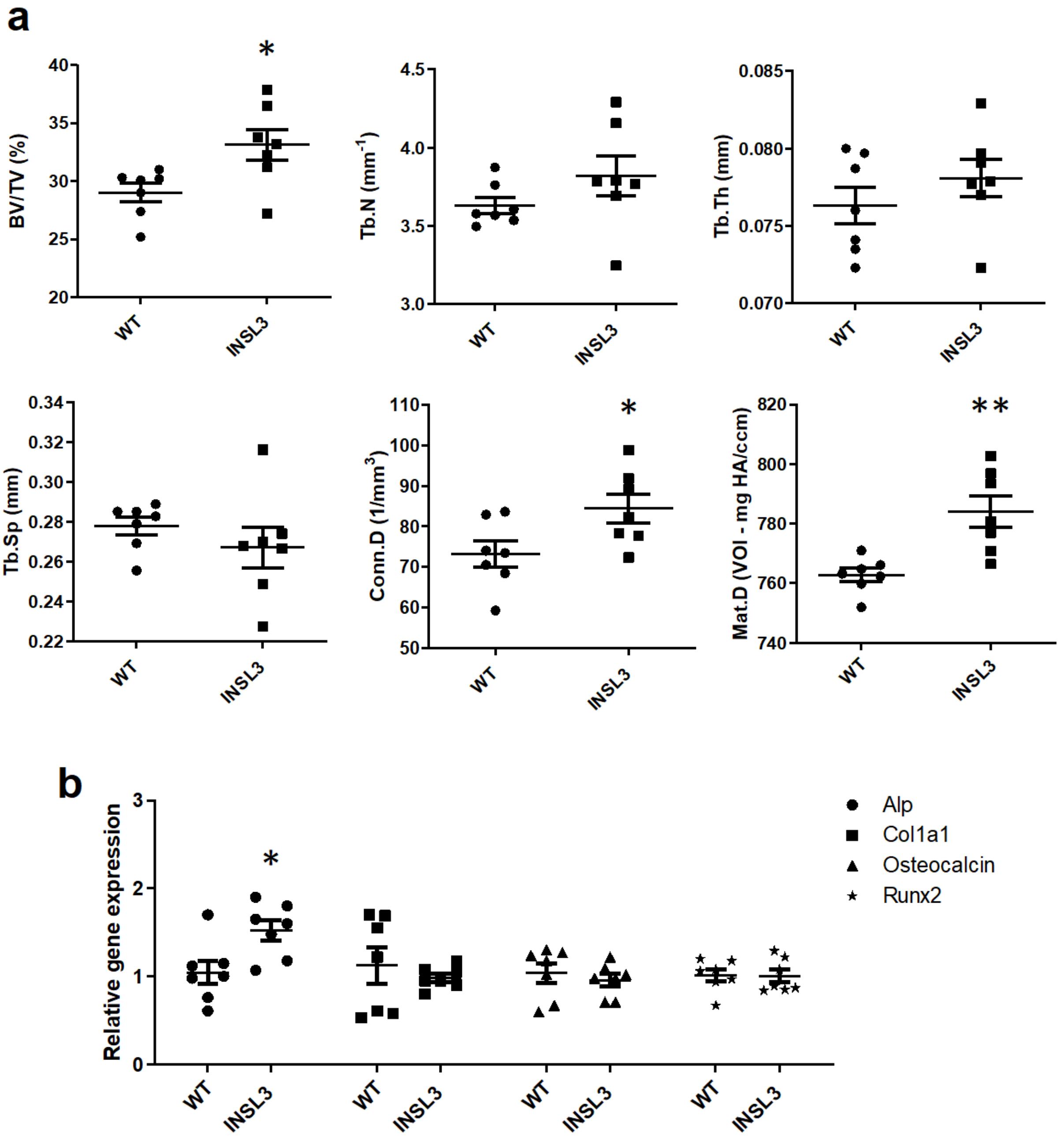

Supplementary Fig. 11 *Insl3* overexpression increases bone formation in female mice. a Trabecular micro-CT parameter in lumbar vertebrae of WT and INSL3 transgenic female mice. **b** Gene expression levels of osteoblast markers in tibias from WT and INSL3 female mice measured by quantitative RT-PCR. Results represent the mean ± SEM of 7 mice per group. *p<0.05, **p<0.01 vs. WT using Student's t-test.

a

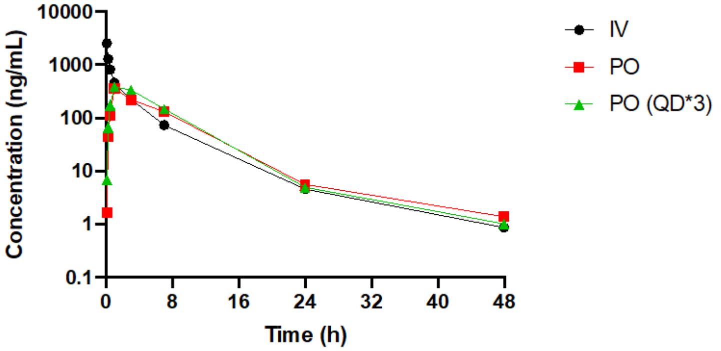

Route	Dosing Level (mg/kg)	Cl_obs (mL/min/kg)	$t_{1/2}$ (h)	t_{max} (h)	C_0 (ng/mL)	AUC_{last} (h*ng/mL)	AUC_{Inf} (h*ng/mL)	AUC_% Extrap (%)	MRT (h)	AUC_{last}/D (h*mg/mL)	V_{ss}_obs (L/kg)	F (%)
IV	3	15.6	6.56	NA	3593	3191	3199	0.258	3.47	1064	3.38	NA
Route	Dosing Level (mg/kg)	Cl_obs (mL/min/kg)	$t_{1/2}$ (h)	t_{max} (h)	C_{max} (ng/mL)	AUC_{last} (h*ng/mL)	AUC_{Inf} (h*ng/mL)	AUC_% Extrap (%)	MRT (h)	AUC_{last}/D (h*mg/mL)	V_{ss}_obs (L/kg)	F (%)
PO	10	NA	3.89	1.00	366	2615	2647	1.20	NA	262	NA	24.8
PO (QD*3)	10	NA	5.88	1.00	389	3261	3269	0.265	NA	326	NA	30.7

b

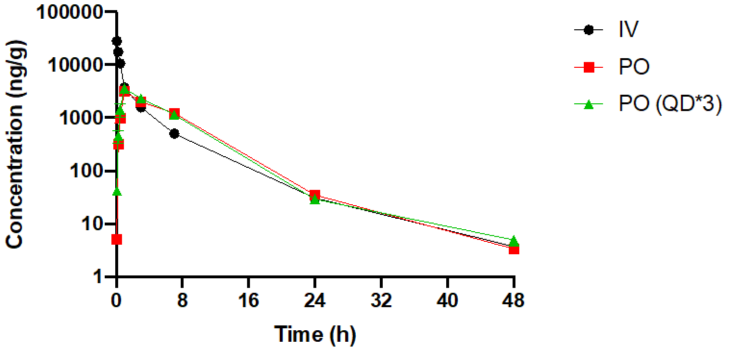

Route	Dosing Level (mg/kg)	t_{max} (h)	C_{max} (ng/g)	AUC_{last} (h*ng/g)	AUC_{Inf} (h*ng/g)	C_{Max} Ratio (Liver/ Plasma)	AUC_{Last} Ratio (Liver/ Plasma)
IV	3	0.083	28040	26492	26524	10.9	8.30
PO	10	1.00	3214	24146	24170	8.78	9.23
PO (QD*3)	10	1.00	3570	24927	24965	9.18	7.64

c

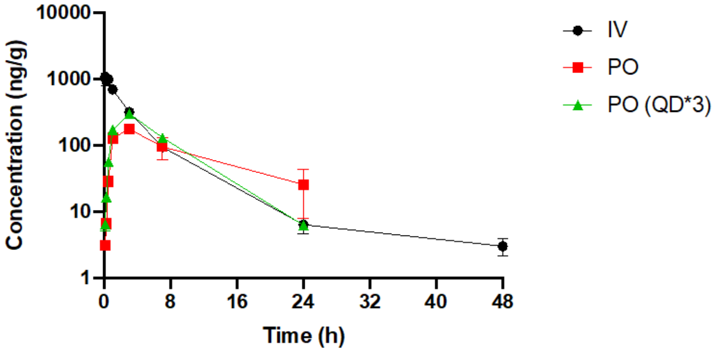

Route	Dosing Level (mg/kg)	t_{max} (h)	C_{max} (ng/g)	AUC_{last} (h*ng/g)	AUC_{Inf} (h*ng/g)	C_{Max} Ratio (Bone/ Plasma)	AUC_{Last} Ratio (Bone/ Plasma)
IV	3	0.083	1072	3650	3687	0.419	1.14
PO	10	3.00	174	1897	2190	0.475	0.725
PO (QD*3)	10	3.000	298	2539	2573	0.766	0.779

Supplementary Fig. 12 Compound 6641 pharmacokinetic study after one 3 mg/kg IV administration, one 10 mg/kg PO administration and three 10 mg/kg PO administrations (QD*3). **a** Plasma, **b** liver and **c** bone profiles in female mice. The actual concentration (ng/g) is the detected value (ng/mL) multiplied by 4. Drug vehicle is 25% aq. 40% HP-b-CD - 75% PEG300. 3 mice were used per time point. Results are expressed as the mean ± SEM.

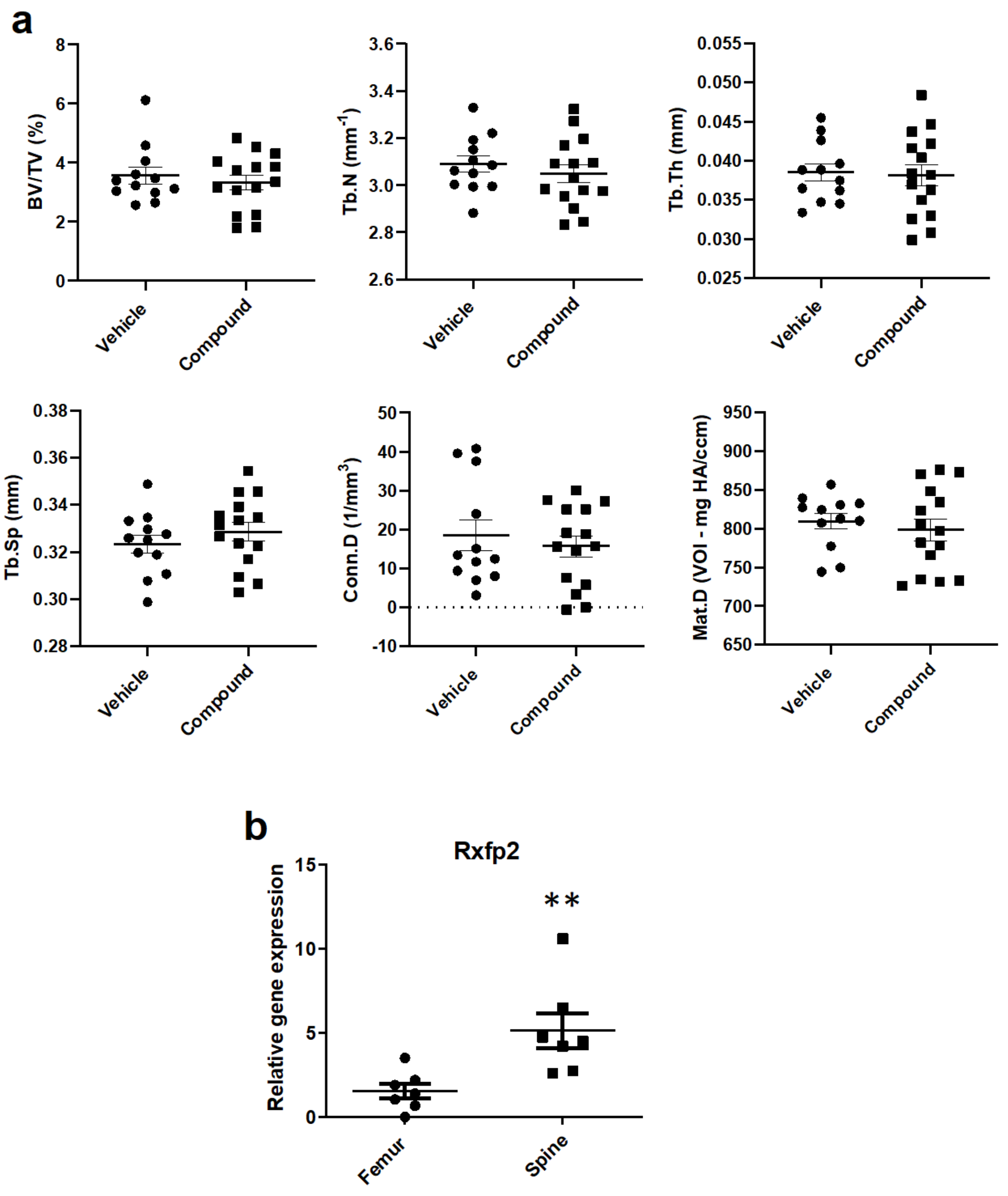

Supplementary Fig. 13 Compound 6641 treatment does not increase bone formation in femur. a Trabecular micro-ct parameters in femur of vehicle- and compound-treated female mice. **b** Gene expression levels of *Rxfp2* in femur and lumbar

spine from untreated female mice measured by quantitative RT-PCR. 12 and 15 mice were used in the vehicle and compound groups respectively. 7 mice were used for gene expression analysis. Results are expressed as the mean ± SEM. **$p<0.01$ vs. femur using Student's t-test.

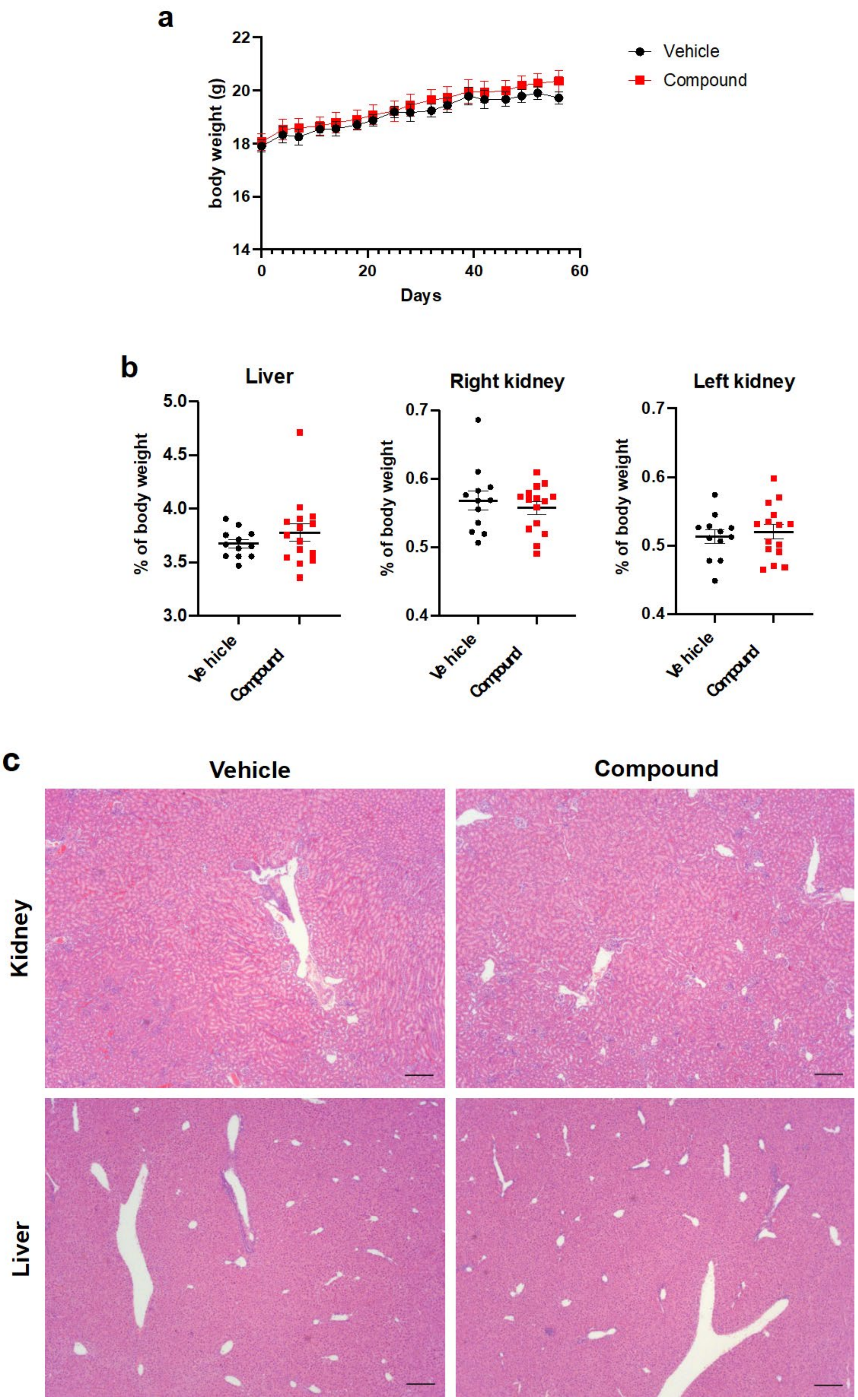
a
body weight (g)
Days
Vehicle
Compound
b
Liver
Right kidney
Left kidney
% of body weight
Vehicle
Compound
c
Vehicle
Compound
Kidney
Liver

Supplementary Fig. 14: Compound 6641 does not show toxicity *in vivo.* a Body weight of all vehicle- and compound-treated mice recorded during the 8-week treatment expressed as the mean ± SEM. **b** Liver and kidney organ weight normalized to body weight at necropsy from all mice expressed as the mean ± SEM. **c** Representative histological sections of liver and kidney from vehicle- and compound-treated mice. Organs from at 3 mice were analyzed per treatment group. Scale 200 µm.

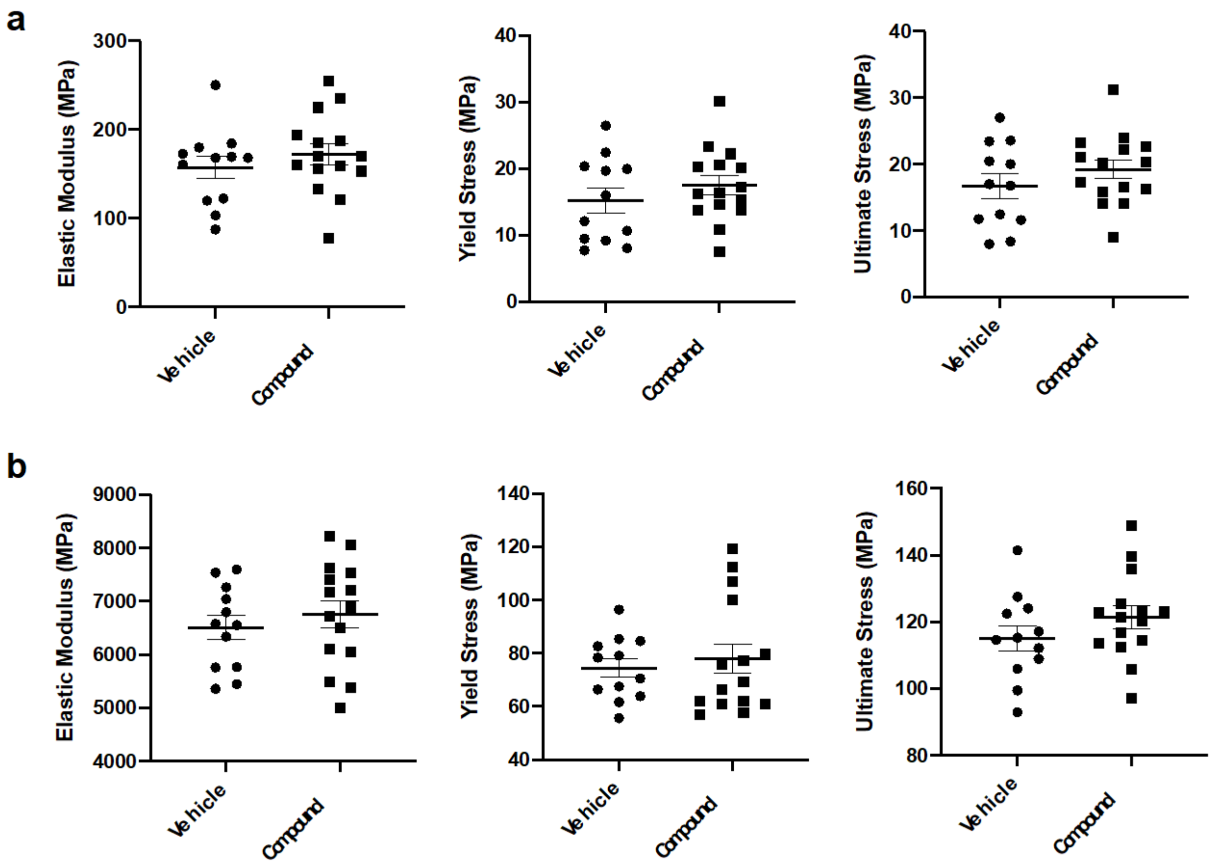

Supplementary Fig. 15: Compound 6641 treatment does not increase bone strength. Biomechanical material properties in **a** vertebrae and **b** femur of vehicle- and compound-treated female mice. 12 and 15 mice were used in the vehicle and compound groups respectively. p = ns (Student's t-test).
