# Supplementary material for: Discovery of small molecule agonists of the Relaxin Family Peptide Receptor 2
Source: Commun Biol. 2022 Nov 4;5:1183. doi: 10.1038/s42003-022-04143-9 (PMC9636434; doi:10.1038/s42003-022-04143-9)
Supplement: Supplementary file 3 — Description of Additional Supplementary Files [file 42003_2022_4143_MOESM3_ESM.pdf]

Description of Additional Supplementary Files

File name: Supplementary Data 1
Description: The results for the primary PRESTO-Tango screen of 320 GPCRs.

File name: Supplementary Data 2
Description: The source data behind the graphs in the main paper and supplemental material.
