# Supplementary material for: Discovery of small molecule agonists of the Relaxin Family Peptide Receptor 2
Source: Commun Biol. 2022 Nov 4;5:1183. doi: 10.1038/s42003-022-04143-9 (PMC9636434; doi:10.1038/s42003-022-04143-9)
Supplement: Supplementary file 6 — Reporting Summary [file 42003_2022_4143_MOESM6_ESM.pdf]

nature portfolio

Corresponding author(s): Alexander Agounik

Last updated by author(s): Sep 26, 2022

Reporting Summary

Nature Portfolio wishes to improve the reproducibility of the work that we publish. This form provides structure for consistency and transparency in reporting. For further information on Nature Portfolio policies, see our Editorial Policies and the Editorial Policy Checklist.

Statistics

For all statistical analyses, confirm that the following items are present in the figure legend, table legend, main text, or Methods section.

n/a	Confirmed	
☐	☒	The exact sample size (*n*) for each experimental group/condition, given as a discrete number and unit of measurement
☐	☒	A statement on whether measurements were taken from distinct samples or whether the same sample was measured repeatedly
☐	☒	The statistical test(s) used AND whether they are one- or two-sided<br>*Only common tests should be described solely by name; describe more complex techniques in the Methods section.*
☒	☐	A description of all covariates tested
☒	☐	A description of any assumptions or corrections, such as tests of normality and adjustment for multiple comparisons
☐	☒	A full description of the statistical parameters including central tendency (e.g. means) or other basic estimates (e.g. regression coefficient) AND variation (e.g. standard deviation) or associated estimates of uncertainty (e.g. confidence intervals)
☐	☒	For null hypothesis testing, the test statistic (e.g. *F*, *t*, *r*) with confidence intervals, effect sizes, degrees of freedom and *P* value noted<br>*Give P values as exact values whenever suitable.*
☒	☐	For Bayesian analysis, information on the choice of priors and Markov chain Monte Carlo settings
☒	☐	For hierarchical and complex designs, identification of the appropriate level for tests and full reporting of outcomes
☒	☐	Estimates of effect sizes (e.g. Cohen's *d*, Pearson's *r*), indicating how they were calculated

Our web collection on statistics for biologists contains articles on many of the points above.

Software and code

Policy information about availability of computer code

Data collection	No software were used
Data analysis	GraphPad, version 9.3.1 was used for all statistical calculations

For manuscripts utilizing custom algorithms or software that are central to the research but not yet described in published literature, software must be made available to editors and reviewers. We strongly encourage code deposition in a community repository (e.g. GitHub). See the Nature Portfolio guidelines for submitting code & software for further information.

Data

Policy information about availability of data

All manuscripts must include a data availability statement. This statement should provide the following information, where applicable:

- Accession codes, unique identifiers, or web links for publicly available datasets
- A description of any restrictions on data availability
- For clinical datasets or third party data, please ensure that the statement adheres to our policy

All data presented in the figures and supplemental material are available in Supplementary Data 2 file.

Human research participants

Policy information about studies involving human research participants and Sex and Gender in Research.

Reporting on sex and gender	N/A
Population characteristics	N/A
Recruitment	N/A
Ethics oversight	N/A

Note that full information on the approval of the study protocol must also be provided in the manuscript.

Field-specific reporting

Please select the one below that is the best fit for your research. If you are not sure, read the appropriate sections before making your selection.

☒ Life sciences ☐ Behavioural & social sciences ☐ Ecological, evolutionary & environmental sciences

For a reference copy of the document with all sections, see nature.com/documents/nr-reporting-summary-flat.pdf

Life sciences study design

All studies must disclose on these points even when the disclosure is negative.

Sample size	All experiments were repeated 3 times with at least 3 replicates
Data exclusions	No data were excluded
Replication	Each experiment was performed with at least 3 biological replicates
Randomization	In animal experiments mice were assigned to control and experimental groups randomly
Blinding	Micro-CT analysis was performed blindly

Reporting for specific materials, systems and methods

We require information from authors about some types of materials, experimental systems and methods used in many studies. Here, indicate whether each material, system or method listed is relevant to your study. If you are not sure if a list item applies to your research, read the appropriate section before selecting a response.

Materials & experimental systems

n/a	Involved in the study	
☐	☒	Antibodies
☐	☒	Eukaryotic cell lines
☒	☐	Palaeontology and archaeology
☐	☒	Animals and other organisms
☒	☐	Clinical data
☒	☐	Dual use research of concern

Methods

n/a	Involved in the study	
☒	☐	ChIP-seq
☐	☒	Flow cytometry
☒	☐	MRI-based neuroimaging

Antibodies

Antibodies used	Antibody: anti-FLAG M1 Ab (F3040, Sigma-Aldrich, Burlington, MA); Alexa Fluor 488 goat anti-mouse IgG (A11001, Invitrogen, Waltham, MA)
Validation	All antibodies were validated by their source company. They have been previously described and used in published studies

Eukaryotic cell lines

Policy information about cell lines and Sex and Gender in Research

Cell line source(s)	HEK293T cells (ATCC CRL-3216) are from ATCC. They were transfected with various mouse and human gene constructs as described in this and previously published studies. Primary human calvarial osteoblast (HCO) cells (ScienCell, Carlsbad, CA).
Authentication	HEK293T cells were authenticated by ATCC. HCO cells were assessed by expression of human osteoblast specific markers and by the company.
Mycoplasma contamination	All cells were tested for mycoplasma contamination using PCR. No contamination was found in any of the cells used in this project
Commonly misidentified lines (See ICLAC register)	HEK

Animals and other research organisms

Policy information about studies involving animals; ARRIVE guidelines recommended for reporting animal research, and Sex and Gender in Research

Laboratory animals	C57BL/6J, males and females, 2-4 month old. INSL3 transgenic mice on FVB background, males and females, 2-4 months old. FVB males, 3-6 month old
Wild animals	Study did not involved wild animals
Reporting on sex	Analysis of compounds effects on male and female embryos was conducted. Analysis of lead compound on bone development in 2-4 month old mice was performed only in females as they have negligible level of circulating INSL3 and thus the possible anabolic effects should be more pronounced.
Field-collected samples	No field collected samples
Ethics oversight	All animal studies were approved under protocols 19-009 and 20-027 by the Florida International University Institutional Animal Care and Use Committee and performed following the NIH Guide for the Care and Use of Laboratory Animals.

Note that full information on the approval of the study protocol must also be provided in the manuscript.

Flow Cytometry

Plots

Confirm that:

☒ The axis labels state the marker and fluorochrome used (e.g. CD4-FITC).

☒ The axis scales are clearly visible. Include numbers along axes only for bottom left plot of group (a 'group' is an analysis of identical markers).

☒ All plots are contour plots with outliers or pseudocolor plots.

☒ A numerical value for number of cells or percentage (with statistics) is provided.

Methodology

Sample preparation	All receptor constructs contain a FLAG epitope at the N-terminus 41. Receptor expression was assessed using a previously described protocol 50. HEK293T cells were harvested in 5 mM EDTA 24 hours after transfection and fixed for 10 minutes in 3.7% formaldehyde/PBS. Cells were then washed twice with surface detection buffer (2% FBS, TBS, 1 mM CaCl2) or permeabilization buffer (surface detection buffer + 0.2% Tween-20) and incubated with 0.5 µg anti-FLAG M1 Ab (F3040, Sigma-Aldrich, Burlington, MA) in surface detection buffer for 1 hour at 4°C. Cells were washed again with surface detection or permeabilization buffer and incubated with 1 µg Alexa Fluor 488 goat anti-mouse IgG (A11001, Invitrogen, Waltham, MA) in surface detection buffer for 20 minutes at 4°C protected from light. Cells were washed one last time and resuspended in surface detection buffer for analysis on an Accuri C6 flow cytometer.
Instrument	Accuri C6 flow cytometer (BD Biosciences, Franklin Lakes, NY)
Software	Software provided with the Accuri C6 flow cytometer was used for analysis
Cell population abundance	The samples were a homogeneous cell line. The entire cell population was analyzed, which was 60-80% of events after gating out debris based on FSS/SSC.

Gating strategy

The intact cell population gate (P1) was drawn based on FSS/SSC. Within P1, an AF488-A histogram with a vertical gate was used to determine positive (R) and negative (L) populations. The vertical gate was drawn based on mock-transfected cells where (R) was less than 1% positive.

☒ Tick this box to confirm that a figure exemplifying the gating strategy is provided in the Supplementary Information.
